# American Medical Journals

**Published:** 1853-03

**Authors:** 


*American Medical Journals.*—The Western Journal of Medicine and Surgery, (No. for Feb. 1853,) contains an interesting editorial article on medical journalizing, suggested by the occasion of noticing a new candidate for favor in that line. Our friend, the writer of the article, will, we know, excuse the liberty which we take in appropriating portions of his remarks for the benefit of our readers, and also as texts for a few original comments.

Speaking of the great number of medical serials at present issued in this country, he asks, "Is it possible that all the journals in existence and contemplated, can live? Are they and will they be supported? We are no prophets, and shall not attempt to foretell what will be the fate of many of our respected contemporaries; but we are sure we are fully warranted in saying, that at the present time their support is most inadequate. Hardly one comes to us in which there are not complaints that subscribers do not pay well. The cover of our own journal bears too frequent testimony to the tardiness with which the demands of our publishers are met. As a body, we take it that the medical editors of the United States labor for a smaller pecuniary remuneration than any other class of men in the community. The hope of such reward cannot be a motive to these enterprises."

It was remarked by Adam Smith, that almost every one, in commencing an undertaking in life, evinces an overweening reliance on his own good fortune. Although multitudes in a similar undertaking have failed, *he* expects to succeed. Perhaps this law of the mind may serve to explain the reiterated efforts to establish medical journals. Were the editorial neophyte governed by the results of previous undertakings of the same sort, he would hardly feel desirous to incur the chances of failure, but he is far from subjecting *his* expectations to the calculation of probabilities. It is fortunate that this egotism, or hopefulness (whichever of these names we choose to give it) is so powerful an element ef human character. Without it undoubtedly there would be still fewer of the relatively small number of undertakings in the different fields of effort which are crowned with success.

Another explanation of the frequent accessions to the list of medical editors is, that the editorial functions are considered to be rather a kind of recreation than otherwise. To write articles, make selections, remodel contributions, and perhaps correct proof, are thought to be duties filling up agreeably the small gaps of time which are at the disposal of the physician. To have such a resource is considered an advantage on the score of relaxation. In this respect disappointment is sure to follow. Novelty may for a brief period give a charm to editorial labors, but ere long they become pleasant only as other labors are so, by habit, and satisfying that want of occupation which Providence has made the condition of happiness in this world.

We find in the article to which we have referred, the following account of the origin of medical journalism in this country, and of the number of medical periodicals now in existence:

"It was near the close of the last century when our first periodical devoted to medicine was established, and at that time there were few such journals in existence anywhere. The *Medical Repository* was begun at New York in 1698, and for fifteen years had no ally or rival in our country. From first to last, in fact, it may be said to have occupied the field alone, for while it went on to the completion of its twenty-third volume, its few contemporaries, after struggling on for a year or two, were discontinued for want of patronage. The *Medical Recorder* succeeded to the popularity of the Medical Repository, and thirty years ago, if not the only one in existence, was the only one that was widely circulated, or had any considerable duration. Another was at length established by its side in Philadelphia, and this still goes on under a new name, the largest, the most elaborate, and the best supported of all our professional serials. The *Boston Medical and Surgical Journal* in age ranks next to the *American Journal of the Medical Sciences*, and its accomplished and amiable editor, we believe, has been longer on the tripod than any of his brethren, and is therefore the patriarch of the fraternity.

"Looking over our exchange list, we find that the number of our medical journals has now swelled to twenty-eight, not to speak of those devoted to Pharmacy, Insanity, Dentistry, and the Sciences allied to medicine, or of that mongrel brood which defiles while professing to reform and advance our noble profession. Of these New York furnishes five, Pennsylvania four, Tennessee four, Ohio two, Louisiana two, Kentucky two, and Massachusetts, New Hampshire, New Jersey, Virginia, South Carolina, Georgia, Missouri, Iowa, and Illinois, each one. All France and Great Britain, together, scarcely do more than this."

On the subject of the literary character of medical journals, our *confrere* makes the following remarks, which, it must be admitted, are founded in fact:

"We have remarked, that most of these publications afford but too indubitable proof of insufficient pecuniary support. Most of them barely pay expenses, if, indeed, they are not a tax upon their conductors; and in this connection another question naturally suggests itself—*is their literary support any better?* We believe that no one, after a careful inspection of the weekly and monthly issues of our medical press, will answer this question in the affirmative. The pages of our journals testify to a sad deficiency of practiced, competent contributors. Any one who will be at the trouble to estimate the proportion of "original" matter in them, one month with another, will find that it is small, and, what is worse, that the articles forming this department have too probably obtained admission, in many cases, for no better reason than the straitness of the editor's supplies. A journal once launched forth must be continued to the end of the year, whether it has subscribers or not, and its pages must be filled with or without the aid of contributors. Publishing day comes round and the editor is not prepared for it; he racks his brains for matter, but discovers, at length, that his powers of production have their limits. His Hippocrene has run dry. In despair he seizes upon whatever may chance to lie within his reach, retouches, or remodels it, vamps it up, and sends it fourth among his "original" essays and cases."

The following extract lets out a secret of editorial experience which, since our contemporary has *peached*, we may confess is no novelty to us. Our readers will be amused with the letter of the repudiating subscriber, and, under the circumstances, they will find no fault with the editor for reproducing the correspondence, *verbatim et literatim*, not attempting, as in other instances, to renovate the composition, so that the author will hardly recognize himself in print. *M. Jeffries, M. D.*, will find his animal the identical one sent to pasture. Inasmuch, however, as the owner himself has *gone to grass*, it is very doubtful if he ever knows that he has been made conspicuous as a writer. But to the extract, which must be read before what has just been written will be intelligible:

"We once heard of a correspondent of a western journal who, on the appearance of his first article in print, wrote a kind letter to the editor, in which he related the following anecdote: He had once a friend, he said, who concluded to buy a little horse at auction because he was going cheap—an ill-formed, starved, sorry looking little creature. He sent him to the country to be recruited. After a few weeks his pony was brought back to him, but so metamorphosed that he did not recognize him. He could hardly be persuaded that the plump, sleek animal before him was the shabb ything that he had a little while before dispatched to the country. We may add that this correspondent was not discouraged by the liberties which the editor took with his manuscript, but has continued to write, and now when his papers return to him he has no difficulty in recognizing them. We have no doubt but the experience of our brethren of the quill would furnish many similar instances. But all correspondents are not so reasonable, nor so improvable. What, for example, could be made of a man who wrote thus?

'Dear Sir I wish you to Stop sending the Medical Journal to me for I will leave for oregon this Spring So close up for it is the no a countest thing I ever read it is at least 50 years behind the times you had better stop grinding or turn Eclectic then you will be a benefit to mankind.

M. JEFFERIES M. D.'

"We think this rare literary specimen is worth preserving, and doubt much whether any of our brethren can furnish from their correspondence anything to match it. *Dr. Jefferies*, it will be remarked, does not deign to make a stop in his letter. It is to be hoped that he did not stop till he got to Oregon."

